# On the Use of the Chimaphila Umbellata in the Treatment of Fungus Articuli, or White Swelling

**Published:** 1846-10

**Authors:** Y. C. Blakey

**Affiliations:** Glasgow, Howard Co. Mo.


*On the use of the Chimaphila Umbellata in the Treatment of Fungus Articuli, or White Swelling.* By Y. C. Blakey, M. D., of Glasgow, Howard Co. Mo.

Under the name of white swelling, several diseases of the joints have been considered as one and the same, but which differ in many respects. Brodie's arrangement, which I consider as good or better than any other, is as follows: 1st. Inflammation of the synovial membrane; 2nd. Morbid change of structure in the synovial membrane; 3d. Ulceration of the cartilages of the joints; 4th. Scrofulous disease of the joints, having its origin in the cancellous structure of the bones. As I do not consider it necessary upon the present occasion to enter into any disquisition, relative to the above forms of this disease, as it respects either the opinions of Brodie or others, I shall merely give the outlines of my treatment of several cases, which entirely relieved the sufferers. The first case, which I shall present nearly verbatim from my own Case Book, is as follows:

*Feb. 5th*, 1844.—Wyatt, aged 17 years, the servant of Mrs. E. was brought to my house in a carriage. On enquiry I found he had been more or less affected about one of his knees for some time, and for several weeks before he was sent to my care, had been attended by a neighboring physician, without any change for the better, but gradually got worse. This boy was of a scrofulous family, as his mother was affected with it for years, and still has more or less swelling in the glands of the neck; and his brother, (there were but two children,) died two years ago with every symptom of phthisis pulmonalis.

When I examined my patient, I found one of his knees three times its natural size, the skin of the leg of an unnatural ashy color, the boy being tolerably black for one of his race; considerable wasting of the limb, pulse 96, and some white fur upon his tongue. I looked upon the case as scrofulous white swelling, and concluded in my own mind there could be little done towards effecting a radical cure, as I had often treated and seen such cases treated, but had never known a cure to follow, but more or less lameness to inevitably succeed all our efforts, if we did not ultimately have to resort to the knife to rescue the sufferer from the grave.

*Feb. 6th.*—The boy disturbed my family last night with his incessant moans, from the excruciating pain he suffered all night. To-night I gave him an anodyne, and after duly weighing his case came to the conclusion to give a trial to the chimaphila umbellata, as the iodine, blisters, setons, &c. &c., had been used already in his case without benefit.

*Feb. 7th.*—I commenced giving my patient the infusion of pipsissewa, a pint to be drank each day. The formula for making it I took from Wood and Bache's Dispensatory, and twice a day, morning and night, I had a fresh poultice made of oat-meal and the infusion, and applied to the whole knee; diet light, and to keep the recumbent position. To-night I gave him another anodyne.

*Feb. 8th.*—Found no difference in the case, and continued up the above course each day until the 14th, when I did not give him his accustomed anodyne; notwithstanding, heard no complaint from its omission. He did not complain sufficiently after this to require any anodynes, but continued gradually to mend, his swelling about the knee to diminish, until the end of March he could walk without a crutch or support of any kind, and on the 15th April I discharged him cured. He remains up to the present time entirely well, and has as much strength in one knee as the other.

*July 10th.*—An old case I had formerly treated by the usual remedies, was brought to me to extract some pieces of bone which had exfoliated; this I accomplished, and commenced the same course with this patient, Mr. A. D., as the boy already described, when after the persevering use of the chimaphila for three or four months, the running from the different openings in the upper portion of the arm, as this patient's swelling was the head of the os humori, closed, and he has not been annoyed by his disease up to the present time. I could present other cases, but as these are sufficient to invite the attention of the profession to the use of this article in the treatment of this horrid disease, the "opprobrium" of our art, I shall leave it to the consideration of my professional brethren, with the hope that I have thrown in a "mite" that will be of benefit to suffering humanity.

